# A Community-Based Restaurant Initiative to Increase Availability of Healthy Menu Options in Somerville, Massachusetts: Shape Up Somerville

**Published:** 2009-06-15

**Authors:** Christina D. Economos, Sara C. Folta, Jeanne Goldberg, Miriam Nelson, David Hudson, Jessica Collins, Zachariah Baker, Eliza Lawson

**Affiliations:** New Balance Chair in Childhood Nutrition, John Hancock Center for Physical Activity and Nutrition, Friedman School of Nutrition Science and Policy; Tufts University, Boston, Massachusetts; Tufts University, Boston, Massachusetts; Tufts University, Boston, Massachusetts; State of Oregon Department of Human Services, Salem, Oregon; Partners for a Healthier Community, Inc, Springfield, Massachusetts; Sustainable Agriculture Coalition and Organic Farming Research Foundation; Rhode Island Department of Health, Providence, Rhode Island

## Abstract

**Background:**

Environmental factors at the community level may play a role in the development and maintenance of obesity. Because many US families frequently eat meals outside of the home, restaurants are an environmental factor that can affect their health. The purpose of this project was to test the feasibility of a community-based restaurant initiative that targets families and young children.

**Context:**

Somerville, Massachusetts, is an ethnically diverse, densely populated city. Approximately 44% of elementary school children in Somerville are overweight or obese. The restaurant initiative described here was conducted as part of a larger community-based environmental intervention, Shape Up Somerville: Eat Smart, Play Hard (SUS), designed to improve energy balance by making small changes in all aspects of a child's environment.

**Methods:**

Restaurant initiative activities were establishing criteria for approval as an SUS restaurant; conducting brief one-on-one interviews with 15 restaurant owners and managers; recruiting restaurants; and monitoring and evaluating restaurants' ability to adhere to the criteria, using questionnaires and site visits.

**Consequences:**

Establishing approval criteria for restaurants required several iterations and ongoing flexibility. Barriers to participation included lack of time and interest and concerns about potential profit losses. The strategy of publicizing approved restaurants facilitated participation in the program. Twenty-eight percent of actively recruited restaurants participated in the initiative. Approximately one-half of restaurants fully complied with all approval criteria.

**Interpretation:**

Despite limited feasibility, the initiative provided valuable visibility and branding of the intervention within the community as well as lessons for working with restaurants to improve health.

## Background

One-third of US children aged 6 to 11 years are overweight or at high risk for becoming overweight (1) and consequently face serious potential consequences to their long-term health and quality of life (2). Environmental factors at the community level may play a role in the development and maintenance of obesity and, therefore, are a natural target for intervention (3). One such factor is the quantity and quality of foods eaten outside the home, particularly at restaurants, which are a major part of most families' lives. The proportion of nutrients obtained from foods outside the home has increased over the past 2 decades (4), paralleling the increase in obesity. Americans spend nearly half of their food dollars on food prepared away from home (5). Several studies have demonstrated that the more frequently an individual eats out, the greater that person's intake of calories, fat, and sodium is (4,6-8). Furthermore, there is some evidence for a relationship between frequency of eating at restaurants and body weight and body fat in both children and adults (6,8,9).

In terms of the role that restaurants may play in obesity, the primary focus has been on chain restaurants, especially fast-food outlets (10). They are a growing segment that represent about half of all restaurant business (10), and they generally have centralized decision making and consistent menus, characteristics that make them good targets for policy intervention. Local, community restaurants have been less frequently targeted and represent a particular challenge, mainly because of their heterogeneity. However, they constitute a substantial proportion of restaurants (10) and have potential to be a synergistic component of community-wide health promotion interventions.

Several programs have attempted to change the community restaurant environment to promote health (11-15). Strategies used in these interventions were to work with restaurants to increase availability of and promote healthier options. To our knowledge, no programs have targeted families and young children. We tested the feasibility of a community restaurant initiative specifically targeted to this demographic. The initiative was conducted as a component of a larger community-based environmental intervention, Shape Up Somerville: Eat Smart, Play Hard (SUS), designed to improve energy balance in elementary school children by making small changes in all aspects of a child's environment (16). Energy balance is defined as a state in which energy intake through the consumption of foods and beverages is approximately equivalent to energy expended. The overall SUS intervention demonstrated an effect on the prevention of undesirable weight gain in the intervention community compared with 2 control communities (16). The goal of the restaurant initiative was to support a healthy environment within the community by working with local restaurants, especially restaurants frequented by families, to increase the availability of healthful alternatives and smaller portions of food.

## Context

We conducted this project in Somerville, Massachusetts, a densely populated city that borders Boston to the north. Somerville occupies 4.1 square miles and has approximately 75,000 residents. Somerville is ethnically diverse, and 29.3% of the total population is foreign born (17). The median household income is $46,315, and 13.0% of families with children under age 18 live below the poverty line (17).

At baseline, 44.4% of elementary school children in Somerville were at or above the 85th percentile for body mass index (16), compared with a national average of 33.3% for children aged 6 to 11 years (1). Formative research for the project included interviews with key informants from the community and focus groups with parents and children. We chose restaurants as one of the SUS intervention points on the basis of the outcomes of this formative research. In Massachusetts, consumers spend approximately $32 million per day on food away from home (18).

## Methods

We conducted the following activities as part of the SUS restaurant initiative: establishing criteria for approval as an SUS restaurant, conducting formative research, recruiting restaurants, and monitoring and evaluating restaurants' ability to adhere to the criteria. We developed the initiative during the spring and summer of 2003 and implemented it as a component of the SUS intervention during the 2003-2004 school year. We monitored it for sustainability during the 2004-2005 school year. The SUS intervention, including the restaurant initiative, was approved by the institutional review board at Tufts University.

### Establishing the "Shape Up Approved" criteria

The initial set of approval criteria for restaurants, which we developed in the spring of 2003, was based on the National School Lunch Program regulations (19) and specified that restaurants offer at least 1 children’s meal that met the guidelines for food-based menu planning. For example, the meal was required to contain a certain amount of meat or meat alternative, fruits and vegetables, and grains. Initial feedback from restaurant owners and managers indicated that these criteria were not feasible and that new criteria should be developed. In response, we established 4 objectives for the new criteria. We determined that criteria must 1) be straightforward, 2) discriminate between restaurants that are offering healthy choices and those that are not offering healthy choices, 3) specify that calories be reduced, and 4) provide visibility and brand awareness for the overall SUS intervention. On the basis of these objectives, a second set of criteria specified the following: restaurants must offer some entrees as half-size portions, some fruits, vegetables, or both as a side dish, and low-fat milk or water (as an alternative to sugar-sweetened beverages); healthier options must be highlighted in some way within the restaurant; and an SUS seal of approval must be displayed in the restaurant window. These criteria are consistent with the recommendations of the Food and Drug Administration’s Keystone Forum on Away-From-Home Foods (10). Our criteria were refined on the basis of results of the formative research, and final criteria are described in the Consequences section.

### Formative research

Our original formative research plan was to conduct 3 focus groups with restaurant owners and managers from different types of restaurants in the community. Research staff attempted to recruit mainly by telephone and by in-person visits. A $20 incentive for participation was offered. However, repeated attempts failed to recruit any participants. Owners and managers indicated that they had very little time and would not participate in a scheduled activity. Therefore, we decided to make unscheduled site visits to restaurants and conduct brief one-on-one interviews. We divided the city into geographic regions, and within each city section we started with an area that had a large number of restaurants that were popular with families based on the information gained from the parent focus groups conducted for the overall SUS intervention. The visits generally took place midmorning or midafternoon, during nonpeak hours, which allowed researchers to speak with owners and managers for 5 to 10 minutes. Research staff described the initiative, obtained feedback on the restaurant owners' and managers' interest in conducting the initiative and the approval criteria, and asked for their perceptions of the benefits and barriers to participation. Research staff kept a log of restaurants visited and wrote notes on the interviews within a day of completing them. Fifteen one-on-one interviews were completed.

### Restaurant recruitment

The recruitment phase started in September of 2003 and lasted through January 2004. Restaurants were prioritized by type and potential for change. Family-friendly sit-down restaurants were given highest priority, followed by delicatessens and sandwich shops. We gave pizza shops lower priority because formative research indicated that the changes necessary to meet the criteria would be least likely to occur in these types of restaurants. Bakeries, pastry shops, coffee shops with only dessert options, and bars or lounges were not recruited because it was considered misleading to promote them as "healthy" places to eat. Large franchise restaurants (including fast-food restaurants) also were not recruited because most decisions related to participation and menu change are made at the corporate level and change at the local franchise level would be extremely difficult. Starting with high-priority restaurants, we first attempted to contact owners and managers by telephone, then mailed them a postcard with program information, and finally conducted in-person visits.

We developed a recruitment kit that initially included a restaurant information guide, SUS contact information, a sample SUS newsletter, and a letter of agreement. The restaurant information guide introduced the overall SUS intervention, described the restaurant initiative, and included a question-and-answer page. The letter of agreement outlined the responsibilities of participating restaurants. Over time, recruitment kits were augmented with media articles about the project and a list of participating restaurants.

"Shape Up Approved" restaurants received a 4-inch window decal (Figure) and laminated signs and table tents listing the "Shape Up Approved" criteria. Restaurants were required to highlight approved meals and items. To help avoid the cost of reprinting menus, owners and managers were given 1-inch stickers that could be placed on existing menus and were given assistance in designing menu inserts. They could also highlight approved items on menu boards or signs.

**Figure.**
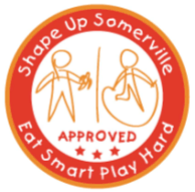
Shape Up Somerville Approved restaurant decal for display in front window or door of participating restaurants, Shape Up Somerville Restaurant Initiative, Somerville, Massachusetts, 2003-2004

### Monitoring and evaluation

To help assess the initiative, we conducted site visits 3 to 6 months after initial approval and obtained written evaluation surveys from the owners and managers. During the site visits, research staff documented compliance with the SUS approval criteria, asked owners and managers about customer reactions to the initiative, and obtained feedback on the program materials (seal, stickers, table tents). As part of the written evaluation surveys, owners and managers were asked to rate their own compliance with criteria, list any changes they had made in their menu as a result of participation, estimate how often customers ordered the healthier menu items and how often they mentioned SUS, rate nutrition awareness as a result of program participation, and state whether program participation had been beneficial to the restaurant.

## Consequences

**Box. Shape Up Somerville Seal of Approval Criteria for Restaurants**

- Must offer:
  - Smaller-sized portions
  - Fruits/vegetables available as side dishes and/or entrees
  - Low-fat or nonfat dairy products (Asian restaurants exempted)
- Must highlight healthier options on a menu board, the menu itself, a laminated sign, or a table tent
- Must display an SUS seal of approval in the restaurant door or window

We further refined the approval criteria on the basis of results of the formative research. Owners and managers expressed considerable concern about half-sized portions because they were not able to offer them at half the price. Waste was also an issue because several items had to be made whole and cut to half size. For example, making a half-size burrito or wrap was problematic because of the wrap size and shape. In keeping with the objective to reduce calories, this criterion was changed from half-sized portions to smaller-sized portions. Owners and managers also had concerns about replacing fries and chips with vegetables, which are expensive and perishable. They agreed to make fruits and vegetables an option in place of fries or chips, usually for an additional charge. The study team felt that this would be a reasonable compromise as long as the option was clearly made known to customers on menus or signs. The criteria were also changed to low-fat dairy instead of low-fat milk. Asian restaurants were exempted from this requirement for cultural reasons. Specifying water as an alternative to soda was unnecessary because all restaurants offered it. The final criteria are listed in the Box.

We learned during the recruitment process that additional flexibility was needed. For example, although offering smaller portion sizes was a criterion, several of the restaurants that were interested in participating were entirely buffet-style. They became SUS approved after agreeing to display a laminated sign reminding customers to eat smaller portion sizes.

During the brief interviews, most owners and managers expressed favorable attitudes about offering healthier options, but many were concerned about the possible effect on profits. They indicated that any publicity resulting from participation would be an attractive incentive. Therefore, we developed a strategy for publicizing the approved restaurants. Publicity included articles and coupons in SUS newsletters (a parent newsletter reaching 811 families and a community newsletter reaching 353 community partners [16]); articles in the Tufts University student newspaper mentioning the approved restaurants (Tufts University borders Somerville to the north); a series of articles in the *Somerville Journal* newspaper titled "Where's Joe?" that spotlighted the mayor of Somerville eating healthy options at approved restaurants; catering opportunities at SUS events, meetings, and trainings; and the creation of a Healthy Meeting Planning Guide listing approved restaurants as catering options. The guide was given to various departments at the City of Somerville, multiple local and community organizations, the Somerville Public Schools, and departments within Tufts University. When recruiting, research staff found owners and managers to be highly receptive to this type of publicity plan.

Twenty-one restaurants became "Shape Up Approved" restaurants. This represents approximately 12% of total restaurants in Somerville (n = 171) and 28% of those that were actively recruited (n = 74). This number included 8 ethnic restaurants (Mexican, Brazilian, Asian, Haitian, and Italian), 5 American-style restaurants, 5 cafés, 1 seafood restaurant, 1 sandwich shop, and 1 pizza shop.

Results from on-site visits showed that, within 6 months of signing the agreement, 10 of 21 restaurants fully complied with all approval criteria. Eleven failed to mark the healthier options, and 1 also failed to display the SUS seal of approval. All met the nutrition criteria. Owners and managers were notified of noncompliance, and follow-up visits were conducted in 4 restaurants approximately 3 months after initial visits. Two restaurants had come into full compliance and 2 had not.

Written evaluation surveys were obtained from 10 of the 21 owners and managers during the initiative (Table). Four of the 10 had made changes to their menus; 6 of the 10 reported that customers ordered "Shape Up Approved" items from their menus at least once per week; and 5 reported that customers asked about or mentioned SUS at least once per week. Seven of 10 believed that it had been beneficial for them to participate in the program, although only 3 indicated that the program had drawn a new base of customers to their restaurants. Seven of 10 indicated that they were more aware of nutrition as a result of participating in the program. Half thought their staff was more aware of nutrition, and 4 of 10 "strongly agreed" or "agreed" that their customers were more aware of nutrition as a result of the program.

## Interpretation

Our results suggest that a community restaurant initiative is feasible, but there are limits to what can be accomplished in this environment. We encountered many barriers to implementation. An overall challenge to the initiative was the lack of a central leadership body to approach; there was no active restaurant association in the community.

Development of the approval criteria for restaurants was difficult and iterative. A key factor in the process was the development of a set of underlying objectives for the criteria. The objectives allowed us to be flexible and respond to restaurant needs while upholding the integrity and purpose of the initiative, which was critical because each restaurant had to be uniquely evaluated with respect to the criteria. Flexibility was also important with respect to conducting the program in a multicultural community. A large number of ethnic restaurants participated in the program, each of which needed to be uniquely evaluated. There were also issues related to cultural acceptability of the changes.

The initial plan to conduct formative research using focus groups was found unfeasible. Convincing busy restaurant owners and managers to participate in focus groups as part of future studies may require a fairly substantial incentive. That was not possible in this project because of budget limitations. Brief, unscheduled interviews with restaurant owners and managers proved to be a more viable approach, although there were limitations. The interviews lacked depth and breadth because of time constraints, and the benefits of group interaction were not realized. However, our findings are consistent with those of a previously reported focus group study with restaurant owners, where lack of time and concerns about revenue loss were reported as major barriers to project participation (20). In our experience, the brief interviews were an effective way to reach this population to obtain the information necessary for development of all aspects of the initiative.

There were also barriers to recruitment. Although site visits were much more effective than recruiting via mail or telephone, we often failed to make contact with the owner or manager. There was a lack of interest in the project, and there were concerns about profitability. In retrospect, it may have been useful to enlist someone with restaurant experience to assist with recruitment. Owners and managers may have been more receptive to someone who they felt had in-depth knowledge of their concerns and constraints. Stressing potential advantages to the restaurant was a critical component of the recruitment strategy. Publicity was a strong incentive, and it was useful to have a concrete plan in place. Another incentive was to be perceived as community-minded and caring about the health of children and families.

There were challenges to the actual implementation of the initiative. Few menu changes occurred because owners and managers viewed alterations to the menu as a potential risk to profits. Restaurants that were SUS-approved were reluctant to make additional changes, and those that did not already meet the nutrition criteria were difficult to recruit. About half of the approved restaurants failed to mark healthier items as specified by the criteria. We attempted to facilitate this by creating stickers, signs, and menu inserts for the restaurants. However, crowded menus, menu boards, and table tops hindered implementation, and owners and managers lacked the time and will to overcome these barriers. All approved restaurants were compliant with the requirement to place the SUS seal of approval on their door or window except 1, suggesting that this aspect of the program was feasible.

About half the owners and managers indicated that customers ordered the SUS-approved items on the menu at least once per week. Restaurants respond to customer demand, and it may be useful in future studies to focus efforts on attempting to create this customer demand within the community.

Despite the many barriers, relatively large research staff effort, and limited feasibility of this project, it was worthwhile. The goal of the overall SUS intervention was to make small changes that affect all aspects of a child's day. Evidence suggests that restaurants are a key component of a child's environment because more children are eating food away from home (4). The SUS intervention was successful at making enough small changes to significantly affect the weight trajectory of elementary school children (16). However, determining the individual contribution of the restaurant initiative to the overall intervention success is not possible. Perhaps one of the greatest benefits of the initiative was that it provided visibility and branding of the intervention within the community, thus creating awareness and synergy with other aspects of the project. The lessons learned in this project will help inform future community-based restaurant projects.

## Figures and Tables

**Table.** Survey Questions and Results, Shape Up Somerville Restaurant Initiative, Somerville, Massachusetts, 2003-2004

**Question**	Response (N = 10)	
Did you make changes to your menu or your way of operating as a result of your participation in Shape Up Somerville?	Yes (n = 4)	No (n = 6)
Has it been beneficial for you to participate in the Shape Up Somerville restaurant program?	Yes (n = 7)	Neutral (n =3)
How often per week do customers place an order for a Shape Up Approved entrée?	1 or more times per week (n = 6)	Less than once per week (n = 4)
How often per week do customers ask about or mention Shape Up Somerville?	1 or more times per week (n = 5)	Less than once per week (n = 5)
I am more aware of nutrition as a result of participating in the Shape Up Somerville program.	Strongly agree/agree (n = 7)	Neutral/disagree^a^ (n = 3)
My staff is more aware of nutrition as a result of the Shape Up Somerville program.	Strongly agree/agree (n = 5)	Neutral/disagree^a^ (n = 5)
The customers are more aware of nutrition as a result of the Shape Up Somerville program.	Strongly agree/agree (n = 4)	Neutral/disagree^a^ (n = 6)
The Shape Up Somerville program has drawn a new base of customers to my restaurant.	Strongly agree/agree (n = 3)	Neutral/disagree^a^ (n = 7)

a Strongly disagree was a response option but was not chosen for any of the questions.
